# The Role of Deep Learning in Advancing Breast Cancer Detection Using Different Imaging Modalities: A Systematic Review

**DOI:** 10.3390/cancers14215334

**Published:** 2022-10-29

**Authors:** Mohammad Madani, Mohammad Mahdi Behzadi, Sheida Nabavi

**Affiliations:** 1 Department of Mechanical Engineering, University of Connecticut, Storrs, CT 06269, USA; 2 Department of Computer Science and Engineering, University of Connecticut, Storrs, CT 06269, USA

**Keywords:** artificial intelligence, breast cancer, deep learning, histopathology, imaging modality, mammography

## Abstract

**Simple Summary:**

Breast cancer is the most common cancer, which resulted in the death of 700,000 people around the world in 2020. Various imaging modalities have been utilized to detect and analyze breast cancer. However, the manual detection of cancer from large-size images produced by these imaging modalities is usually time-consuming and can be inaccurate. Early and accurate detection of breast cancer plays a critical role in improving the prognosis bringing the patient survival rate to 50%. Recently, some artificial-intelligence-based approaches such as deep learning algorithms have shown remarkable advancements in early breast cancer diagnosis. This review focuses first on the introduction of various breast cancer imaging modalities and their available public datasets, then on proposing the most recent studies considering deep-learning-based models for breast cancer analysis. This study systemically summarizes various imaging modalities, relevant public datasets, deep learning architectures used for different imaging modalities, model performances for different tasks such as classification and segmentation, and research directions.

**Abstract:**

Breast cancer is among the most common and fatal diseases for women, and no permanent treatment has been discovered. Thus, early detection is a crucial step to control and cure breast cancer that can save the lives of millions of women. For example, in 2020, more than 65% of breast cancer patients were diagnosed in an early stage of cancer, from which all survived. Although early detection is the most effective approach for cancer treatment, breast cancer screening conducted by radiologists is very expensive and time-consuming. More importantly, conventional methods of analyzing breast cancer images suffer from high false-detection rates. Different breast cancer imaging modalities are used to extract and analyze the key features affecting the diagnosis and treatment of breast cancer. These imaging modalities can be divided into subgroups such as mammograms, ultrasound, magnetic resonance imaging, histopathological images, or any combination of them. Radiologists or pathologists analyze images produced by these methods manually, which leads to an increase in the risk of wrong decisions for cancer detection. Thus, the utilization of new automatic methods to analyze all kinds of breast screening images to assist radiologists to interpret images is required. Recently, artificial intelligence (AI) has been widely utilized to automatically improve the early detection and treatment of different types of cancer, specifically breast cancer, thereby enhancing the survival chance of patients. Advances in AI algorithms, such as deep learning, and the availability of datasets obtained from various imaging modalities have opened an opportunity to surpass the limitations of current breast cancer analysis methods. In this article, we first review breast cancer imaging modalities, and their strengths and limitations. Then, we explore and summarize the most recent studies that employed AI in breast cancer detection using various breast imaging modalities. In addition, we report available datasets on the breast-cancer imaging modalities which are important in developing AI-based algorithms and training deep learning models. In conclusion, this review paper tries to provide a comprehensive resource to help researchers working in breast cancer imaging analysis.

## 1. Introduction

Breast cancer is the second most fatal disease in women and is a leading cause of death for millions of women around the world [1]. According to the American Cancer Society, approximately 20% of women who have been diagnosed with breast cancer die [2,3]. Generally, breast tumors are divided into four groups: normal, benign, in situ carcinoma, and invasive carcinoma [1]. A benign tumor is an abnormal but noncancerous collection of cells in which minor changes in the structure of cells happen, and they cannot be considered cancerous cells [1]. However, in situ carcinoma and invasive carcinoma are classified as cancer [4]. In situ carcinoma remains in its organ and does not affect other organs. On the other hand, invasive carcinoma spreads to surrounding organs and causes the development of many cancerous cells in the organs [5,6]. Early detection of breast cancer is a determinative step for treatment and is critical to avoiding further advancement of cancer and its complications [7]. There are several well-known imaging modalities to detect and treat breast cancer at an early stage including mammograms (MM) [8], breast thermography (BTD) [9], magnetic resonance imaging (MRI) [10], positron emission tomography (PET) [11], computed tomography (CT) [11], ultrasound (US) [12], and histopathology (HP) [13]. Among these modalities, mammograms (MMs) and histopathology (HP), which involve image analysis of the removed tissue stained with hematoxylin and eosin to increase visibility, are widely used [14,15]. Mammography tries to filter a large-scale population for initial breast cancer symptoms, while histopathology tries to capture microscopic images with the highest possible resolution to find exact cancerous tissues at the molecular level [16,17]. In practice for breast cancer screening, radiologists or pathologists observe and examine breast images manually for diagnosis, prognosis, and treatment decisions [7]. Such screening usually leads to over- or under-treatment because of inaccurate detection, resulting in a prolonged diagnosis process [18]. It is worth noting that only 0.6% to 0.7% of cancer detections in women during the screening are validated and 15–35% of cancer screening fails due to errors related to the imaging process, quality of images, and human fatigue [19,20,21]. Several decades ago, computer-aided detection (CAD) systems were first employed to assist radiologists in their decision-making. CAD systems generally analyze imaging data and other cancer-related data alone or in combination with other clinical information [22]. Additionally, based on the statistical models, CADs can provide results about the probability of diseases such as breast cancer [23]. CAD systems have been widely used to help radiologists in patient care processes such as cancer staging [23]. However, conventional CAD systems, which are based on traditional image processing techniques, have been limited in their utility and capability.

To tackle these problems and enhance efficiency as well as decrease false cancer detection rates, precise automated methods are needed to complement the work of humans or replace them. AI is one of the most effective approaches capturing much attention in analyzing medical imaging, especially for the automated analysis and extraction of relevant information from imaging modalities such as MMs and HPs [24,25]. Many available AI-based tools for image recognition to detect breast cancer have exhibited better performance than traditional CAD systems and manually examining images by expert radiologists or pathologists due to the limitations of current manual approaches [26]. In other words, AI-based methods avoid expensive and time-consuming manual inspection and effectively extract key and determinative information from high-resolution image data [26,27]. For example, a spectrum of diseases is associated with specific features, such as mammographic features. Thus, AI can learn these types of features from the structure of image data and then detect the spectrum of the disease assisting the radiologist or histopathologist. It is worth noting that in contrast to human inspection, algorithms are mainly similar to the black box and cannot understand the context, mode of collection, or meaning of viewed images, resulting in the problem of “shortcut” learning [28,29]. Thus, building interpretable AI-based models is necessary. AI models can generally be categorized into two groups to interpret and extract information from image data: (1) Traditional machine learning algorithms which need to receive handcrafted features derived from raw image data as preprocessing steps. (2) Deep learning algorithms that process raw images and try to extract features by mathematical optimization and multiple-level abstractions [30]. Although both approaches have shown promising results in breast cancer detection, recently, the latter approach has attracted more interest mainly because of its capability to learn the most salient representations of the data without human intervention to produce superior performance [31,32]. This review assesses and compresses recent datasets and AI-based models, specifically created by deep learning algorithms, used on TBD, PET, MRI, US, HP, and MM in breast cancer screening and detection. We also highlight the future direction in breast cancer detection via deep learning. This study can be summarized as follows: (1) Review of different imaging modalities for breast cancer screening. (2) Comparison of different deep learning models proposed in the most recent studies and their achieved performances on breast cancer classification, segmentation, detection, and other analysis. (3) Lastly, the conclusion of the paper and suggestions for future research directions. The main contributions of this paper can be listed as follows:

1. We reviewed different imaging tasks such as classification, segmentation, and detection through deep learning algorithms, while most of the existing review papers focus on a specific task.
2. We covered all available imaging modalities for breast cancer analysis in contrast to most of the existing studies that focus on single or two imaging modalities.
3. For each imaging modality, we summarized all available datasets.
4. We considered the most recent studies (2019–2022) on breast cancer imaging diagnosis employing deep learning models.

## 2. Imaging Modalities and Available Datasets for Breast Cancer

In this study, we summarize well-known imaging modalities for breast cancer diagnosis and analysis. As many existing studies have shown, there are several imaging modalities, including mammography, histopathology, ultrasound, magnetic resonance imaging, positron emission tomography, digital breast tomosynthesis, and a combination of these modalities (multimodalities) [10,32,33]. There are various public or private datasets for these modalities. Approximately 70% of available public datasets are related to mammography and ultrasound modalities demonstrating the prevalence of these methods, especially mammography, for breast cancer screening [31,32]. On the other hand, the researcher also widely utilized other modalities such as histopathology and MRI to confirm cancer and deal with difficulties related to mammography and ultrasound imaging modalities such as large variations in the image’s shape, morphological structure, and the density of breast tissues, etc. Here, we outline the aforementioned imaging modalities and available datasets for breast cancer detection.

### 2.1. Mammograms (MMs)

**Figure 1:**
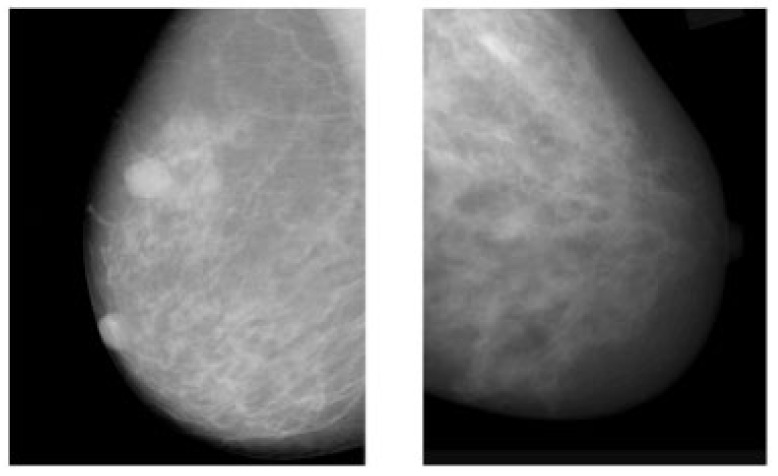
Example of breast cancer images using traditional film MMs. Reprinted/adapted with permission from [49]. 2021, Elsevier.

The advantages of mammograms, such as being cost-effective to detect tumors in the initial stage before development, mean that MMs are the most promising imaging screening technique in clinical practice. MMs are generally images of breasts produced by low-intensity X-rays (Figure 1) [33]. In this imaging modality, cancerous regions are brighter and more clear than other parts of breast tissue, helping to detect small variations in the composition of the tissues; therefore, it is used for the diagnosis and analysis of breast cancer [34,35] (Figure 1). Although MMs are the standard approach for breast cancer analysis, it is an inappropriate imaging modality for women with dense breasts [36], since the performance of MMs highly depends on specific tumor morphological characteristics [36,37]. To deal with this problem, using automated whole breast ultrasound (AWBU) or other methods are suggested with MMs to produce a more detailed image of breast tissues [38]. 

For various tasks in breast cancer analysis, such as breast lesion detection and classification, MMs are generally divided into two forms: screen film mammograms (SFM) and digital mammograms (DMM). DMM is widely categorized into three categories consisting of full-field digital mammograms (FFDM), digital breast tomosynthesis (DBT), and contrast-enhanced digital mammograms (CEDM) [39,40,41,42,43,44]. SFM was the standard imaging method in MMs because of its high sensitivity (100%) in the analysis and detection of lesions in breasts composed primarily of fatty tissue [45]. However, it has many drawbacks, including the following: (1) SFM imaging needs to be repeated with a higher radiation dose because some parts of the image in SFM have lesser contrast and cannot be further improved, and (2) various regions of the breast image are represented according to the characteristic response of the SFM [19,45]. Since 2010, DMM has replaced film as the primary screening modality. The main advantages of digital imaging over file systems are the higher contrast resolution and the ability to enlarge the image or change the contrast and brightness. These advantages help radiologists to detect subtle abnormalities, particularly in a background of dense breast tissue, more easily. Most studies comparing digital and film mammography performance have found little difference in cancer detection rates [46]. Digital mammography increases the chance of detecting invasive cancer in premenopausal and perimenopausal women and women with dense breasts. However, it increases false-positive findings as well [46]. Randomized mammographic trials/randomized controlled trials (RMT/RCT) represent the most important usage of MMs, through which large-scale screening for breast cancer analysis is performed. Despite the great capability of MMs for early-stage cancer detection, it is difficult to use MMs alone for detection. Because it requires additional screening tests along with mammographic trials/RMT such as breast self-examination (BSE) and clinical breast examination (CBE), which are more feasible methods to detect breast cancer at early stages to improve breast cancer survival [38,47,48]. Additionally, BSE and CBE avoid tremendous harm due to MMs screening, such as repeating the imaging process. More details about the advantages and disadvantages of MMs are provided in Table 1.

### 2.2. Digital Breast Tomosynthesis (DBT)

**Figure 2:**
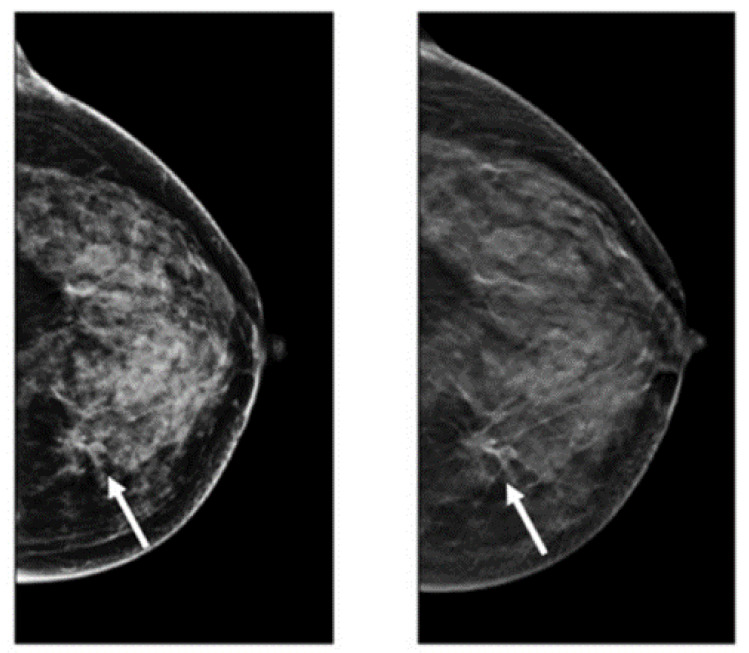
Images of cancerous breast tissue by DBT imaging modality [61]. Reprinted/adapted with permission from [61]. 2021, Elsevier.

DBT is a novel imaging modality making 3D images of breasts by the utilization of X-rays captured from different angles [50]. This method is similar to what is performed in mammograms, except the tube with the X-ray moves in a circular arc around the breast [51,52,53] (Figure 2). Repeated exposures to the breast tissue at different angles produce DBT images in half-millimeter slices. In this method, computational methods are utilized to collect information received from X-ray images to produce z-stack breast images and 2D reconstruction images [53,54]. In contrast to the conventional FSM method, DBT can easily cover the imaging of tumors from small to large size, especially in the case of small lesions and dense breasts [55]. However, the main challenging issue regarding the DBT is the long reading time because of the number of mammograms, the z-stack of images, and the number of recall rates for architectural distortion type of breast cancer abnormality [56]. After FFDM, DBT is the commonly used method for imaging modalities. Many studies recently used this imaging modality for breast cancer detection due to its favorable sensitivity and accuracy in screening and producing better details of tissue in breast cancer [57,58,59,60]. Table 1 provides details of the pros and cons of DBT for breast cancer analysis.

### 2.3. Ultrasound (US)

**Figure 3:**
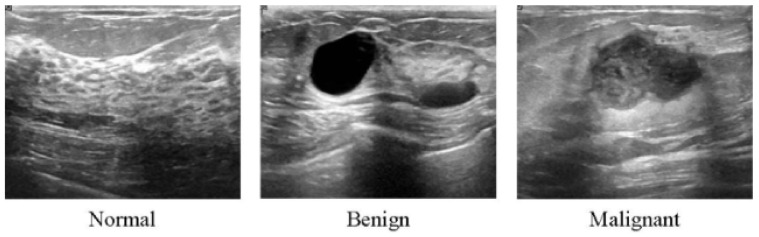
Ultrasound images from breast tissue for normal, benign, and malignant [80].

All of the aforementioned modalities can endanger patients and radiologists because of possible overdosage of ionizing radiation, making these approaches slightly risky and unhealthy for certain sensitive patients [62]. Additionally, these methods show low specificity, meaning the low ability to correctly determine a tissue without disease as a negative case. Therefore, although the aforementioned imaging modalities are highly used for early breast cancer detection, the US as a safe imaging modality has been used [62,63,64,65,66,67] (Figure 3). Compared to MMs, the US is a more convenient method for women with dense breasts. It is also useful to characterize abnormal regions and negative tumors detected by MMs [68]. Some studies showed the high accuracy of the US in detecting and discriminating benign and malignant masses [69]. US images are used in three broad combinations, i.e., (i) simple two-dimensional grayscale US images, (ii) color US images with shear wave elastography (SWE) added features, and (iii) Nakagami colored US images without any need for ionizing radiation [70,71]. It is worth noting that Nakagami-colored US images are responsible for the region of interest extraction by better detection of irregular masses in the breast. Moreover, US can be used as a complement to MMs owing to its availability, inexpensiveness compared to other modalities, and it being well tolerated by patients [70,72,73]. In a recent retrospective study, US breast imaging has shown high predictive value when combined with MMs images [74]. US images, along with MMs, improved the overall detection by about 20% and decreased unnecessary biopsy tasks by 40% in total [67]. Moreover, US is a reliable and valuable tool for metastatic lymph node screening in breast cancer patients. It is a cheap, noninvasive, easy-to-handle and cost-effective diagnostic method [75]. However, the US represents some limitations. For instance, the interpretation of US images is highly difficult and needs an expert radiologist to comprehensively understand these images. This is because of the complex nature of US images and the presence of speckle noise [76,77]. To deal with this issue, new technologies have been introduced in breast US imaging, such as automated breast ultrasound (ABUS). ABUS produces 3D images using wider probes. Shin et al. [78] improved how ABUS allows more appropriate image evaluation for large breast masses compared to conventional breast US. On the other hand, ABUS showed the lowest reliability in the prediction of residual tumor size and pCR (pathological complete response) [79]. Table 1 highlights more details about the weaknesses and strengths of the US imaging modality.

### 2.4. Magnetic Resonance Imaging (MRI)

**Figure 4:**
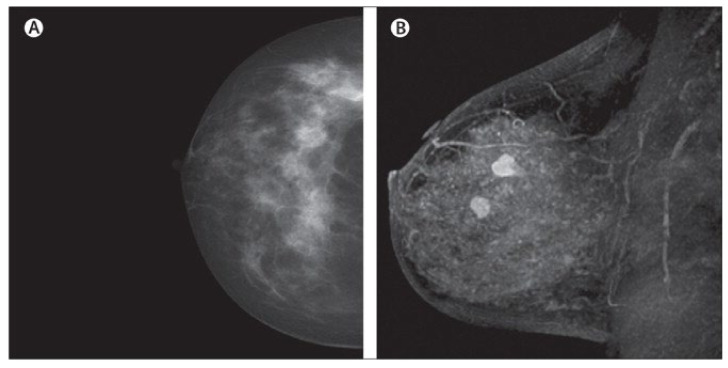
Dense cancerous breast tissue images conducted by MRI method from different angles. (**A**) Normal; (**B**) malignant [82]. Reprinted/adapted with permission from [82]. 2011, Elsevier.

MRI creates images of the whole breast and presents it as thin slices that cover the entire breast volume. It works based on radio frequency absorption of nuclei in the existence of potent magnetic fields. MRI uses a magnetic field along with radio waves to capture multiple breast images at different angles from a tissue [81,82,83] (Figure 4). By the combination of these images together, clear and detailed images of tissues are produced. Hence, MRI creates much clearer images for breast cancer analysis than other imaging modalities [84]. For instance, the MRI image shows many details clearly, leading to easy detection of lesions that are considered benign in other imaging modalities. Additionally, MRI is the most favorable method for breast cancer screening in women with dense breasts without any ionizing and other health risks, which we have seen in other modalities such as MMs [85,86]. Another interesting issue about MRI is its capability for producing high-quality images with a clearer view via the utilization of a contrast agent before taking MRI images [87,88]. Furthermore, MRI is more accurate than MM, DBT, and the US in evaluating residual tumors and predicting pCR [79,89], which helps clinicians to select appropriate patients for avoiding surgery after neoadjuvant chemotherapy (first-line treatment of breast cancer) when pCR is obtained [90,91]. Even though MRI exhibits promising advantages, such as high sensitivity, it shows low specificity, and it is time consuming and expensive, especially since its reading time is long [92,93]. It is worth noting that some new MRI-based methods, such as ultrafast breast MRI (UF-MRI), create much more efficient images with high screening specificity with short reading time [94,95]. Additionally, diffusion-weighted MR imaging (DWI-MRI) and dynamic contrast-enhanced MRI (DCE-MRI) provide higher volumetric resolution for better lesion visualization and lesion temporal pattern enhancement to use in breast cancer diagnosis and prognosis and correlation with genomics [53,81,96,97,98]. Details about various MRI-based methods and their pros and cons are available in Table 1.

### 2.5. Histopathology

**Figure 5:**
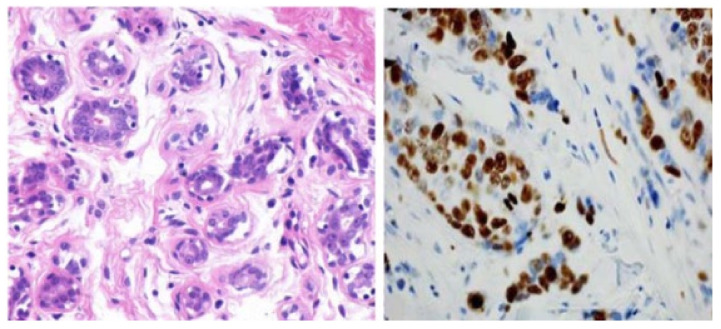
Images of the breast from H&E (haemotoxylin and eosin) stained image of a benign case provided by histopathology imaging modality [105]. Reprinted/adapted with permission from [105]. 2017, Elsevier.

Recently, various studies have confirmed that the gold standard for confirmation of breast cancer diagnosis, treatment, and management is given by the histopathological analysis of a section of the suspected area by a pathologist [99,100,101]. Histopathology consists of examining tissue lesion samples stained, for example, with hematoxylin and eosin (H&E) to produce colored histopathologic (HP) images for better visualization and detailed analysis of tissues [102,103,104] (Figure 5). Generally, HP images are obtained from a piece of suspicious human tissue to be tested and analyzed by a pathologist [105]. HP images are defined as gigapixel whole-slide images (WSI) from which some small patches are extracted to enhance the analysis of these WSI (Figure 5). In other words, pathologists try to extract small patches related to ROI from WSI to diagnose breast cancer subtypes, which is a great advantage of HPs, enabling them to classify multiple classes of breast cancer [106,107] for prognosis and treatment. Additionally, much more meaningful ROI can be derived from HPs, in contrast to other imaging modalities confirming outstanding authenticity for breast cancer classification, especially breast cancer subtype classification. Furthermore, one of the most important advantages of HPs is their capability to integrate multi-omics features to analyze and diagnose breast cancer with high confidence [108]. TCGA is the most favorable resource for breast histopathological images. The TCGA database is widely employed in multi-level omics integration investigations. In other words, within TCGA, HPs provide contextual features to extract morphological properties, while molecular information from omics data at different levels, including microRNA, CNV, and DNA methylation [108], are also available for each patient. Integrating morphology and multiomics information provides an opportunity to more accurately detect and classify breast cancer. Despite these advantages, HPs have some limitations. For example, analyzing multiple biopsy sections, such as converting an invasive biopsy to digital images, is a lengthy process requiring a high concentration level due to the cell structures’ microscopic size [109]. More drawbacks and advantages of the HP imagining modality are summarized in Table 1.

### 2.6. Positron Emission Tomography (PET)

**Figure 6:**
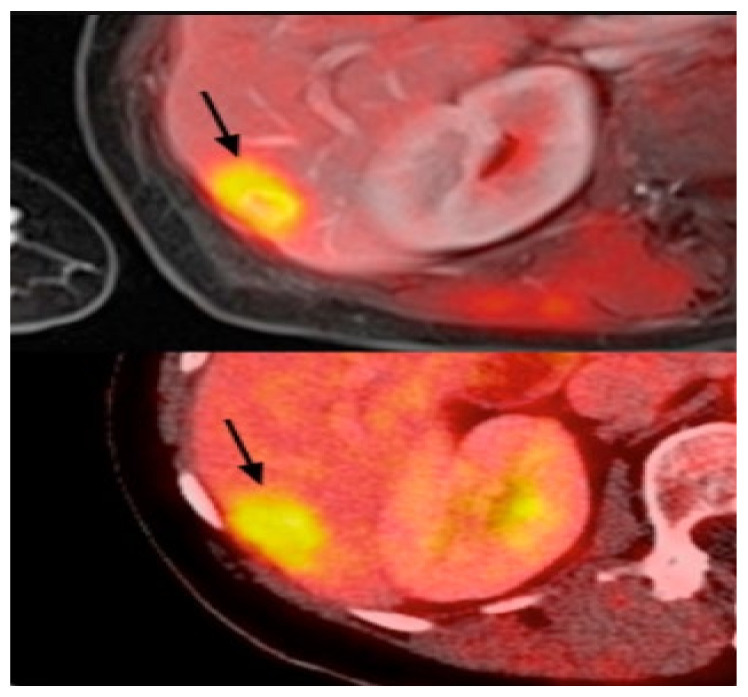
Example of PET images for breast cancer analysis [118]. Reprinted/adapted with permission from [118]. 2021, Elsevier.

PET uses radiotracers for visualizing and measuring the changes in metabolic processes and other physiological activities, such as blood flow, regional chemical composition, and absorption. PET is a recent effective imaging method showing the promising capability to measure tissues’ in vivo cellular, molecular, and biochemical properties (Figure 6). One of the key applications of PET is the analysis of breast cancer [110]. Studies highlighted that PET is a handy tool in staging advanced and inflammatory breast cancer and evaluating the response to treatment of the recurrent disease [34,35]. In contrast to the anatomic imaging method, PET highlights a more specific targeting of breast cancer with a larger margin between tumor and normal tissue, representing one step forward in cancer detection besides anatomic modalities [111,112,113]. Thus, the PET approach is used in hybrid modalities with CT for specific organ imaging to encourage the advantages of PET and improve spatial resolution, which is one of this modality’s strengths. Additionally, PET uses the integration of radionuclides with some elements or pharmaceutical compounds to form radiotracers, improving the performance of PET [114]. Fluorodeoxyglucose (FDG), a glucose analog, is most commonly used for most breast cancer imaging studies as an effective radiotracer developed for PET imaging [115]. Recent studies clarified a specific correlation between the degree of FDG uptake and several phenotypic features containing a tumor histologic type and grade, cell receptor expression, and cellular proliferation [116,117]. These correlations lead to making the FDG-PET system for breast cancer analysis such as diagnosis, staging, re-staging, and treatment response evaluation [111,118,119]. Another PET system is a breast-dedicated high-resolution PET system designed in a hanging breast imaging modality. Some studies demonstrate that these PET-based modalities can detect almost all breast lesions and cancerous regions [120]. Table 1 summarizes some of PET-based imaging modalities’ limitations and advantages. Also, in Table 2, we provided most commonly used public datasets for different imaging modalities in breast cancer detection.

## 3. Artificial Intelligence in Medical Image Analysis

Artificial intelligence (AI) has become very popular in the past few years because it adds human capabilities, e.g., learning, reasoning, and perception, to the software accurately and efficiently, and as a result, computers gain the ability to perform tasks that are usually carried out by humans. The recent advances in computing resources and availability of large datasets, as well as the development of new AI algorithms, have opened the path to the use of AI in many different areas, including but not limited to image synthesis [121], speech recognition [122,123] and engineering [124,125,126]. AI has been also employed in healthcare industries for applications such as protein engineering [127,128,129,130], cancer detection [131], and drug discovery [132,133]. More specifically, AI algorithms have shown an outstanding capability to discover complex patterns and extract discriminative features from medical images, providing higher-quality analysis and better quantitative results efficiently and automatically. AI has been a great help for physicians in imaging-related tasks, i.e., disease detection and diagnosis, to accomplish more accurate results [134]. Deep learning (DL) [30] is part of a broader family of AI which imitates the way humans learn. DL uses multiple layers to gain knowledge, and the complexity of the learned features increases hierarchically. DL algorithms have been applied in many applications, and in some of them, they could outperform humans. DL algorithms have also been used in various categories in the realm of cancer diagnosis using cancer images from different modalities, including detecting cancer cells, cancer type classification, lesion segmentation, etc. To learn more about DL, we refer interested readers to [135].

### 3.1. Benefits of Using DL for Medical Image Analysis

Comparing the healthcare area with others, it is safe to say that the decision-making process is much more crucial in healthcare systems than in other areas since it directly affects people’s lives. For example, a wrong decision by a physician in diagnosing a disease can lead to the death of a patient. Complex and constrained clinical environments and workflows make the physician’s decision-making very challenging, especially for image-related tasks since they require high visual perception and cognitive ability [136]. In these situations, AI can be a great tool to decrease the false-diagnosis rates by extracting specific and known features from the images or even helping the physician by giving an initial guess for the solution. Nowadays, more and more healthcare providers are encouraged to use AI algorithms due to the availability of computing resources, advancement in image analysis tools, and the great performance shown by AI methods.

### 3.2. Deep Learning Models for Breast Cancer Detection

This section briefly discusses the deep learning algorithms applied to images from each breast cancer modality.

#### 3.2.1. Digital Mammography and Digital Breast Tomosynthesis (MM-DBT)

**Figure 7:**
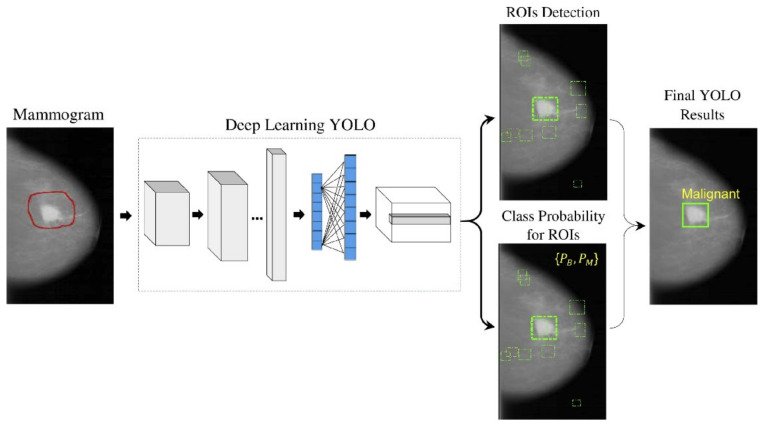
Schematic diagram of the proposed YOLO-based CAD system in [131]. Reprinted/adapted with permission from [131]. 2021, Elsevier.

With the recent technology developments, MM images follow the same trend and take more advanced forms, e.g., digital breast tomosynthesis (DBT). Each MM form has been widely used for breast cancer detection and classification. One of the first attempts to use deep learning for MMs was carried out by [137]. The authors in [137] used a convolutional neural network (CNN)-based model to learn features from mammography images before feeding them to a support vector machine (SVM) classifier. Their algorithm could achieve 86% AUC in lesion classification, which had about 6% improvement compared to the best conventional approach before this paper. Following [137], more studies [138,139,140] have also used CNN-based algorithms for lesion classification. However, in these papers, the region of interest was extracted without the help of a deep learning algorithm, i.e., by employing traditional image processing methods [139] or by an expert [140]. More specifically, the authors in [138] first divided MM images into patches and extracted the features from the patches using a conventional image-processing algorithm, and then used the random forest classifier to choose good candidate patches for their CNN algorithm. Their approach could achieve an AUC of 92.9%, which is slightly better than the baseline method based on a conventional method with an AUC of 91%. With the advancement in DL algorithms and the availability of complex and powerful DL architectures, DL methods have been used to extract ROIs from full MM images. As a result, the input to the algorithm is no longer the small patches, and the full MM image could be used as input. For example, the proposed method in [131] uses YOLO [141], a well-known algorithm for detection and classification, to simultaneously extract and classify ROIs in the whole image. Their results show that their algorithm performs similarly to a CNN model trained on small patches with an AUC of 97%. Figure 7 shows the overall structure of the proposed model in [131].

To increase the accuracy of cancer detection, DBT has emerged as a predominant breast-imaging modality. It has been shown that DBT increases the cancer detection rate (CDR) while decreasing recall rates (RR) when compared to FFDM [142,143,144]. Following the same logic, some DL algorithms have been proposed to apply to DBT images for cancer detection [145,146,147,148,149]. For instance, the authors in [150] proposed a deep learning model based on ResNet architecture to classify the input images into normal, benign, high-risk, or malignant. They trained the model on an FFDM dataset, then fine-tuned the model using 2D reconstruction of DBT images obtained by applying the 2D maximum intensity projection (MIP) method. Their method achieved an AUC of 84.7% on the DBT dataset. A deep CNN has been developed in [145] that uses DBT volumes to classify the masses. Their proposed approach obtained an AUC of 84.7%, which is about 2% higher than the current CAD method with hand-crafted features.

Although deep learning models perform very well in medical image analysis, their major bottleneck is the thirst for training datasets. In the medical field, collecting and labeling data is very expensive. Some studies used transfer learning to overcome this problem. In the study by [151], the authors developed a two-stage transfer learning approach to classify DBT images as mass or normal. In the first stage, the authors fine-tuned a pretrained AlexNet [152] using FFDM images, and then the fine-tuned model was used to train a model using DBT images. The CNN model in the second stage was used as the feature extractor for DBT images, and the random forest classifier was used to classify the extracted features as mass or normal. They obtained an AUC of 90% on their test dataset. In another work in [153], the authors used a VGG19 [154] network trained on the ImageNet dataset as a feature extractor for FFDM and DBT images for malignant and benign classification. The extracted features were fed to an SVM classifier to estimate the probability of malignancy. Their method obtained an AUC of 98% and 97% on the DBT images in CC and MLO view, respectively. These methods show that by using a relatively small training dataset and employing transfer learning techniques, deep learning models can perform well. Most of the aforementioned studies compare their DL algorithms with traditional CAD methods. However, the best way to evaluate the performance of a DL method is to compare that with a radiologist directly. For example, the performance of DL systems on FFDM and DBT has been investigated in [155]. The study shows that a DL system can achieve comparable sensitivity as radiologists in FFDM images while decreasing the recall rate. Additionally, on DBT images, an AI system can have the same performance as radiologists, although the recall rate has increased. 

Table 3 shows the list of recent DL-based models used for MM and DBT with their performances. The application of DL in breast cancer detection is not limited to mammography images. In the following section, we discuss the DL application in other breast cancer imaging modalities. 

#### 3.2.2. Ultrasound (US)

**Figure 8:**
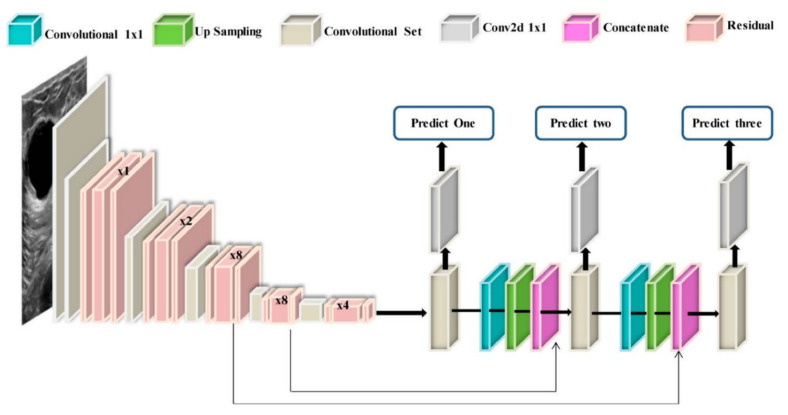
Example of a model architecture for breast cancer subtypes classification from US images via CNN models [222].

As has been explained in Section 2, ultrasound performs much better in detecting cancers and reduces unnecessary biopsy operations [183]. Therefore, it is not surprising to see that the researchers use this type of image in their DL models for cancer detection [184,185,186]. For instance, a GoogleNet [187]-based CNN has been trained on the suspicious ROIs of US images in [184]. The proposed method in [184] achieved an AUC of 96%, which is 6% higher than the CAD-based method with hand-crafted features. The authors in [188,189,190] trained CNN models directly with whole US images without extracting the ROIs. For example, the authors in [190] combined VGG19 and ResNet152 and trained the ensemble network on US images. Their proposed method achieved an AUC of 95% on a balanced, independent test dataset. Figure 8 represents an example of CNN models for breast cancer subtype classification. 

In comparison with datasets for mammography images, there are fewer datasets for US images, and they usually contain much fewer images. Therefore, most of the proposed DL models use some kind of data augmentation method, such as rotation, to increase the size of training data and improve the model performance. However, one should be careful about how to augment US images since some augmentation may decrease the model performance. For example, it has been shown in [186] that performing the image rotation or shift in the longitudinal direction can affect the model performance negatively. The generative adversarial networks (GANs) can also be used to generate synthetic US images with or without tumors [191]. These images can be added to the original training images to improve the model’s accuracy.

The US images have also been used in lesion detection in which, when given an image, the CAD system decides whether the lesion is present. One of the challenges that the researcher faces in this type of problem with normal US images is that there is a need for a US doctor to manually select the images that have lesions for the models. This depends on the doctors’ availability and is usually expensive and time-consuming. It also adds human errors to the system [192]. To solve this problem, a method has been developed in [193] to detect the lesions in real time during US scanning. Another type of US imaging is called the 3D automated breast US scan, which captures the entire breast [194,195]. The authors in [195] developed a CNN model based on VGGNet, ResNet [196], and DenseNet [197] networks. Their approach obtained an AUC of 97% on their private dataset and an AUC of 97.11% on the breast ultrasound image (BUSI) dataset [80].

Some methods combined the detection and classification of lesions in US images in one step [198]. An extensive study in [199] compares different DL architectures for US image detection and classification. Their results show that the DenseNet is a good candidate for classification analysis of US images, which provides accuracies of 85% and 87.5% for full image classification and pre-defined ROIs, respectively. The authors in [200] developed a weakly supervised DL algorithm based on VGG16, ResNet34, and GoogleNet trained using 1000 unannotated US images. They have reported an average AUC of 88%.

Some studies validate the performance of DL algorithms [201,202,203] using expert inference, showing that DL algorithms can greatly help radiologists. This is mostly in cases where the lesion was already detected by an expert, and the DL model is used to classify them. However, unlike the mammography studies, most of the studies are not validated by multiple physicians and do not show the generalizability of their method on multiple datasets which should be addressed in future validations. Table 4 shows the list of recent algorithms used for US images and their performances.

#### 3.2.3. Magnetic Resonance Imaging (MRI)

As explained in Section 2, MRI has higher sensitivity for breast cancer detection in dense breasts [223] than MM and US images. However, the big difference between MRI and MM or US images is that the MRI is a 3D scan, but MM and US are 2D images. Moreover, MRI sequences are captured over time, increasing the MRI dimensionality to 4D (dynamic contrast-enhanced (DCE)-MRI). This makes MRI images more challenging for DL algorithms compared to MM and US images, as most of the current DL algorithms are built for 2D images. One way to address this challenge is to convert the 3D image to 2D by either dividing 3D MRIs into 2D slices [224,225] or using MIP to build a 2D representation [226]. Moreover, most DL algorithms have been developed for colored images, which are 3D images whose third dimension represents the color channels. However, the MRIs are grayscale images. Therefore, some developed MRI models put three consecutive slices of grayscale MRI together and build a 3D image [227,228]. Some other approaches modify the current 2D DL architecture to make them appropriate for MRI 3D scans [229].

**Figure 9:**
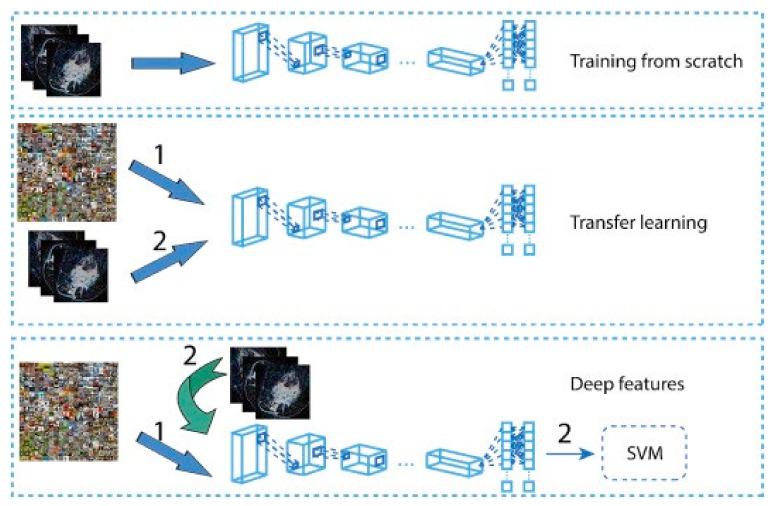
A model architecture for cancer subtypes prediction via ResNet 50 and CNN models from MRI images [237]. Reprinted/adapted with permission from [237]. 2019, Elsevier.

All the above approaches have been used in lesion classification DL algorithms. For example, [230] uses 2D slices of the ROIs as input to their CNN model. They obtained an accuracy of 85% on their test dataset. The MIP technique is used in [231] which obtained an AUC of 89.5%. In the study carried out by Zhou et al. [229], the authors put the grayscale MRIs together and built 3D images for their DL methods. Their algorithm obtained an AUC of 92%. In another study presented in [193], the proposed algorithm uses the actual 3D MRI scans obtaining an AUC of 85.9% by the 3D version of DenseNet [197]. It is worth mentioning that the performance of 2D and 3D approaches cannot be compared since they used different datasets. However, some studies compared their proposed methods with radiologists’ interpretations [228,229]. Figure 9 shows a schematic of a framework for cancer subtype classification with MRI.

Like in MM and US images, the DL methods have been widely used in lesion detection and segmentation problems in MRI images. A CNN algorithm based on RetinaNet [232] has been developed in [233] for detecting lesions from the 4D MR scans. Their method obtained a sensitivity of 95%. One study [234] used a mask-guided hierarchical learning (MHL) framework for breast tumor segmentation based on U-net architecture. Their method achieved the Dice similarity coefficient (DSC) of 0.72 for lesion segmentation. In another work [235], the authors proposed a U-net-based CNN model called 3TP U-net for the lesion segmentation task. Their algorithm obtained a Dice similarity coefficient of 61.24%. Alternatively, the authors in [236] developed a CNN-based segmentation model by refining the U-net architecture to segment the lesions in MRIs. Their proposed method achieved a Dice similarity coefficient of 86.5%. It has to be noted that in most lesion segmentation algorithms, there is a need for a mask that shows the pixels that belong to the breast as ground truth for training. These masks can help the models to focus on the right place and ignore the areas that do not have any information. Table 5 shows the list of recent algorithms used for MRI images and their performances.

#### 3.2.4. Histopathology

In contrast to other modalities, histopathology images are colored images that are provided either as the whole-slide images (WSI) or the extracted image patches from the WSI, i.e., ROIs that are extracted by pathologists. The histopathology images are a great means of diagnosing breast cancer types that are impossible to find with radiology images, i.e., MRIs. Moreover, these images have been used to detect cancer subtypes because of the details they have about the tissue. Therefore, they are widely used with DL algorithms for cancer detection. For example, Ref. [258] employed a CNN-based DL algorithm to classify the histopathology images into four classes: normal tissue, benign lesion, in situ carcinoma, and invasive carcinoma. They combined the classification results of all the image patches to obtain the final image-wise classification. They also used their model to classify the images into two classes, carcinoma, and non-carcinoma. An SVM has been trained on the features extracted by a CNN to classify the images. Their method obtained an accuracy of 77.8% on four-class classification and an accuracy of 83.3% on binary classification. In another work proposed in [259], two CNN models were developed, one for predicting malignancy and the other for predicting malignancy and image magnification levels simultaneously. They used images of size 700 × 460 with different magnification levels. Their average binary classification for benign/malignant is 83.25%. A novel framework was proposed in [260] that uses a hybrid attention-based mechanism to classify histopathology images. The attention mechanism helps to find the useful regions from raw images automatically.

**Figure 10:**
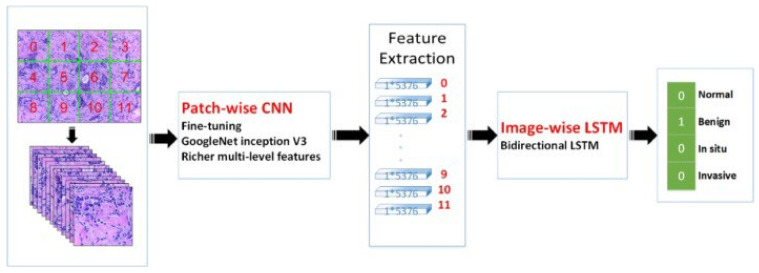
Prediction of breast cancer grades from extracted patches from histopathology images via patch-wise LSTM architecture [264]. Reprinted/adapted with permission from [264]. 2019, Elsevier.

The transfer learning approach has also been employed in analyzing histopathology images since the histopathology image datasets suffer from the lack of a large amount of data required for deep learning models. For example, the method developed in [261] uses pretrained Inception-V3 [187] and Inception-ResNet-V2 [262] and fine-tunes them for both binary and multiclass classification on histology images. Their approach obtained an accuracy of 97.9% in binary classification and an accuracy of 92.07% in the multi-classification task. In another work [263], the authors developed a framework for classifying malignant and benign cells that extracted the features from images using GoogleNet, VGGNet, and ResNet and then combined those features to use them in the classifier. Their framework obtained an average accuracy of 97%. The authors in [264] used a fine-tuned GoogleNet to extract features from the small patches of pathological images. The extracted features were fed to a bidirectional long short-term memory (LSTM) layer for classification. Their approach obtained an accuracy of 91.3%. Figure 10 shows the overview of the method proposed in [264]. GANs have also been combined with transfer learning to further increase classification accuracy. In work carried out in [265], StyleGAN [266] and Pix2Pix [267] were used to generate fake images. Then, VGG-16 and VGG-19 were fine-tuned to classify images. Their proposed method achieved an accuracy of 98.1% in binary classification.

Histopathology images have been widely used for nuclei detection and segmentation. For instance, in the work presented in [268], a novel framework called HASHI was developed that automatically detects invasive breast cancer in the whole slide images. Their framework obtained a Dice coefficient of 76% on their independent test dataset. In the other work performed in [269] for nuclei detection, a series of handcrafted features and features extracted from CNN were combined for better detection. The method used three different datasets and obtained an F-score of 90%. The authors in [270] presented a fully automated workflow for nuclei segmentation in histopathology images based on deep learning and the morphological properties extracted from the images. Their workflow achieved an accuracy and F1-score of 95.4% and 80.5%, respectively. In another work by [271], the authors first extracted the small patches from the high-resolution whole slides, then each small patch was segmented using a CNN along with an encoder-decoder; finally, to combine the local segmentation result, they used an improved merging strategy based on a fully connected conditional random field. Their algorithm obtained a segmentation accuracy of 95.6%. Table 6 shows the performance of recently developed DL methods in histology images.

#### 3.2.5. Positron Emission Tomography (PET)/Computed Tomography (CT)

PET/CT is a nuclear medicine imaging technique that helps increase the effectiveness of detecting and classifying axillary lymph nodes and distant staging [272]. However, they have trouble detecting early-stage breast cancer. Therefore, it is not surprising that PET/CT is barely used with DL algorithms. However, PET/CT has some important applications that DL algorithms can be applied. For example, as discussed in [273], breast cancer is one of the reasons for most cases of bone metastasis. A CNN-based algorithm was developed in [274] to detect breast cancer metastasis on whole-body scintigraphy scans. Their algorithm obtained 92.5% accuracy in the binary classification of whole-body scans.

In the other application, PET/CT can be used to quantify the whole-body metabolic tumor volume (MTV) to reduce the labor and cost of obtaining MTV. For example, in the work presented in [275], a model trained on the MTV of lymphoma and lung cancer patients is used to detect the lesions in PET/CT scans of breast cancer patients. Their algorithm could detect 92% of the measurable lesions.

**Table 6:** The summary of the studies that used histopathology datasets.

Paper	Year	Task	Model	Dataset	Evaluation
Zainudin et al. [276]	2019	Breast Cancer Cell Detection/Classification	CNN	MITOS	Acc = 84.5% TP = 80.55% FP = 11.6%
Li et al. [277]	2019	Breast Cancer Cell Detection/Classification	Deep cascade CNN	MITOSIS AMIDA13 TUPAC16	MITOSIS: F-score = 56.2% AMIDA13: F-score = 67.3% TUPAC16: F-score = 66.9%
Das et al. [278]	2019	Breast Cancer Cell Detection/Classification	CNN	MITOS ATYPIA14	MITOS: F1-score = 84.05% ATYPIA14: F1-score = 59.76%
Gour et al. [279]	2020	Classification	CNN	BreakHis	Acc = 92.52% F1 score = 93.45%
Saxena et al. [280]	2020	Classification	CNN	BreakHis	Avg. Acc = 88%
Hirra et al. [281]	2021	Classification	DBN	DRYAD	Acc = 86%
Senan et al. [282]	2021	Classification	CNN	BreakHis	Acc = 95% AUC = 99.36%
Zewdie et al. [283]	2021	Classification	CNN	Private BreakHis Zendo	Binary Acc = 96.75% Grade classification Acc = 93.86%
Kushwaha et al. [284]	2021	Classification	CNN	BreakHis	Acc = 97%
Gheshlaghi et al. [285]	2021	Classification	Auxiliary Classifier GAN	BreakHis	Binary Acc = 90.15% Sub-type classification Acc = 86.33%
Reshma et al. [286]	2022	Classification	Genetic Algorithm with CNN	BreakHis	Acc = 89.13%
Joseph et al. [287]	2022	Classification	CNN	BreakHis	Avg. Multiclass Acc = 97%
Ahmad et al. [288]	2022	Classification	CNN	BreakHis	Avg. Binary Acc = 99% Avg. Multiclass Acc = 95%
Mathew et al. [289]	2022	Breast Cancer Cell Detection/Classification	CNN	ATYPIA MITOS	F1 score = 61.91%
Singh and Kumar [290]	2022	Classification	Inception ResNet	BHI BreakHis	BHI: Acc = 85.21% BreakHis: Avg. Acc = 84%
Mejbri et al. [291]	2019	Tissue-level Segmentation	DNNs	Private	U-Net: Dice = 86%, SegNet: Dice = 87%, FCN: Dice = 86%, DeepLab: Dice = 86%
Guo et al. [292]	2019	Cancer Regions Segmentation	Transfer learning based on Inception-V3 and ResNet-101	Camelyon16	IOU = 80.4% AUC = 96.2%
Priego-Torres et al. [271]	2020	Tumor Segmentation	CNN	Private	Acc = 95.62% IOU = 92.52%
Budginaitė et al. [293]	2021	Cell Nuclei Segmentation	Micro-Net	Private	Dice = 81%
Pedersen et al. [294]	2022	Tumor Segmentation	CNN	Norwegian cohort [295]	Dice = 93.3%
Khalil et al. [296]	2022	Lymph node Segmentation	CNN	Private	F1 score = 84.4% IOU = 74.9%

## 4. Discussion

Breast cancer plays a crucial role in the mortality of women in the world. Cancer detection in its early stage is an essential task to reduce mortality. Recently, many imaging modalities have been used to give more detailed insights into breast cancer. However, manual analysis of these imaging modalities with a huge number of images is a difficult and time-consuming task leading to inaccurate diagnoses and an increased false-detection rate. Thus, to tackle these problems, an automated approach is needed. The most effective and reliable approach for medical image analysis is CAD. CAD systems have been designed to help physicians to reduce their errors in analyzing medical images. A CAD system highlights the suspicious features in images (e.g., masses) and helps radiologists to reduce false-negative readings. Moreover, CAD systems usually detect more false features than true marks, and it is the radiologist’s responsibility to evaluate the results. This characteristic of CAD systems increases the reading time and limits the number of cases that radiologists can evaluate. Recently, the advancement of AI, especially DL-based methods, could effectively speed up the image analysis process and help radiologists in early breast cancer diagnosis.

Considering the importance of DL-based CAD systems for breast cancer detection and diagnosis, in this paper, we have discussed the applications of different DL algorithms in breast cancer detection. We first reviewed the imaging modalities used for breast cancer screening and diagnosis. Besides a comprehensive discussion, we discussed the advantage and limitations of each imaging modality and summarize the public datasets available for each modality with the links to the datasets. We then reviewed the recent DL algorithms used for breast imaging analysis along with the detail of their datasets and results. The studies presented promising results from DL-based CAD systems. However, the DL-based CAD tools still face many challenges that prohibit them from clinical usage. Here, we discussed some of these challenges as well as the future direction for cancer detection studies.

One of the main obstacles to having a robust DL-based CAD tool is the cost of collecting medical images. The medical images used for DL algorithms should contain reliable annotated images from different patients. Data collection would be very costly for sufficient abnormal data compared to normal cases since the number of abnormal cases is much lower than the normal cases (e.g., several abnormal cases per thousand patients in the breast cancer screening population). The data collection also depends on the number of patients that takes a specific examination and the availability of equipment and protocols in different clinical settings. For example, MM datasets are usually very large datasets, including thousands of patients. However, the MRI or PET/CT datasets contain much fewer patients. Due to the existence of a large public dataset for MM, much more DL algorithms have been developed and validated for the MM modality than other datasets. One way to create a big dataset for different image modalities is multi-institutional collaboration. The dataset obtained from these collaborations covers a large group of patients with different characteristics, different imaging equipment, and clinical settings and protocols. These datasets make the DL algorithms more robust and reliable.

Currently available medical image datasets usually contain a small amount of data. On the other hand, employing DL and exploiting its capabilities on a small amount of training data is challenging. Because the DL algorithms should be trained on a large dataset to have a good performance. Some possible solutions can help to overcome the problems related to small datasets. For example, the datasets from different medical centers can be combined to create a bigger one. However, there are usually some patient privacy policies that should be addressed. Another solution to this problem is using federated learning [297] in which the algorithm is trained on datasets locally, but it should travel between the centers and be trained on the datasets in each center. The federated learning algorithms are not popular yet, and they are not widely implemented. In most cases, the training data cannot be publicly shared; therefore, there is no way to evaluate the DL methods and regenerate the results in the studies. Many studies used transfer learning to overcome the problem of small datasets. Some of the studies used a pre-trained model to extract features from the medical images and then, they used the extracted features to train a DL model for target tasks. However, other studies initialized their model with pre-trained model weights and then fine-tuned their models with the medical image datasets. Although transfer learning shows some improvement for the small datasets, the performance of the target model highly depends on the difference between the characteristics of source datasets and target datasets. In these cases, a negative transfer [298] may occur in which the source domain reduces the learning performance in the target domain. Some studies used data augmentation rather than transfer learning to increase the size of the dataset artificially and improve the model performance. However, one should note that augmenting data does not introduce the independent features to the model; therefore, it does not provide much new knowledge for the DL model compared to new independent images.

The shortage of datasets with comprehensive and fully labeled/annotated data is also another challenge that DL-based CAD systems face. Most of the DL methods are supervised algorithms, and they need fully labeled/annotated datasets. However, creating a large fully annotated dataset is a very challenging task since annotating medical images is time-consuming and may have human errors. To avoid the need for annotated datasets, some papers used unsupervised algorithms, but they obtained less accurate results compared to supervised algorithms.

Another important challenge is the generalizability of the DL algorithms. Most of the proposed approaches work on the datasets obtained with specific imaging characteristics and cannot be used for the datasets obtained from different populations, different clinical settings, or different imaging equipment and protocols. This is an obstacle to the wide use of AI methods in cancer detection in medical centers. Each health clinic should design and conduct a testing protocol for DL-based CAD systems using the data obtained from the local patient population before any clinical usage of these systems. During the testing period, the user should find the weaknesses and strengths of the system based on the output of the system for different input cases. The user should know that what is the characteristics of the failed and correct output and recognize when the system makes mistake and when it works fine. This testing procedure not only evaluates DL-based CAD models but also teaches the user the best way to use DL-based CAD systems.

Another limitation can be the interpretability of DL algorithms. Most DL algorithms are like a black box, and there are no suitable explanations for the decision, and feature selection happens during the training and learning processes. Radiologists usually do not prefer these uninterpretable DL algorithms because they need to understand the physical meaning of the decisions taken by the algorithms and which parts of images are highly discriminative. Recently, some DL-based algorithms such as DeepSHAP [299] were introduced to define an interpretable model to give more insight into the decision-making of DL algorithms in medical image analysis. Therefore, to increase physicians’ confidence and reliability of the decision made by DL tools, the utilization of interpretable approaches and proper explanation of DL algorithms is required for breast cancer analysis, helping widely used DL technology in clinical care applications such as breast cancer analysis.

DL algorithms show outstanding performance in analyzing imaging data. However, as discussed, there are still many challenges that they face. Besides DL algorithms, some studies show that using omics data instead of imaging data may lead to higher classification accuracy [108,300]. The omics data contain fewer but more effective features than imaging data. Moreover, the DL methods may extract the features from the images that are not relevant to the final label and those features may decrease the model performance. On the other hand, processing omics data is more expensive than image processing. Moreover, there are much more algorithms available for image processing than omics processing. Additionally, there are much more imaging data available than omics data.

## 5. Conclusions

Cancer detection in its early stage can improve the survival rate and reduce mortality. The rapid developments in deep learning-based techniques in medical image analysis algorithms along with the availability of large datasets and computational resources made it possible to improve breast cancer detection, diagnosis, prognosis, and treatment. Moreover, due to the capability of deep learning algorithms particularly CNNs, they have been very popular among the research community. In this research, comprehensive detail of the most recently employed deep learning methods is provided for different image modalities in different applications (e.g., classification, and segmentation). Despite outstanding performance by deep learning methods, they still face many challenges that should be addressed before deep learning can eventually influence clinical practices. Besides the challenges, ethical issues related to the explainability and interpretability of these systems need to be considered before deep learning can be expanded to its full potential in the clinical breast cancer imaging practice. Therefore, it is the responsibility of the research community to make the deep learning algorithms fully explainable before considering these systems as decision-making candidates in clinical practice.

## Figures and Tables

**Table 1:** Advantages and limitations of various imaging modalities.

Imaging Modalities	Advantages	Limitations
MM	More than 70% of studies (computational and experimental) for breast cancer analysis. Time- and cost-effective approach for image capturing and processing compared to other modalities No need for highly professional radiologists for diagnosis and cancer detection compared to other methods	Cannot capture micro-calcification because MMs are created via low-dose X-ray Limited capability for diagnosis of cancer in dense breasts Needs more testing for accurate diagnosis Needs various pre-processing for classification because of considering many factors and structures such as the border of the breast, fibrous strands, hypertrophied lobules, etc. which may cause misunderstanding Problems in the visualization of cancer in high breast density
US	A very efficient approach in reducing false negative rates for diagnosis because of its capability in capturing images from different views and angles. A highly safe and most efficient approach for a routine checkup because the US is a non-invasive method Ability to detect invasive cancer areas Highly recommended for the identification of breast lesion ROI because of its additional features such as color-coded SWE images	Capturing low-quality images for examination of the larger amount of tissues Difficult to understand SWE images Single Nakagami parametric image cannot detect cancerous tissues Proper ROI estimation is very difficult because of the shadowing effect making the tumor contour unclear
MRI	Safe method due to no exposure to harmful ionizing radiation Captures images with more detail Captures more suspicious areas for further analysis compared to other modalities Can be improved by adding contrast agents to represent images with more details	Misses some tumors but can be used as a complement of MMs Increases body temperature May lead to some allergies Invasive method and dangerous
HP	Produces color-coded images that help to detect cancer subtypes and early detection of cancer Widely used in cancer diagnosis similar to MMs Shows tissues in two forms including WSI and ROI extracted from WSI Provides more reliable results for diagnosis than any other imaging modalities ROI increases accuracy of cancer diagnosis and analysis Can be stored for future analysis	Expensive and time-consuming method to analyze and need Highly expert pathologist It is tedious to extract ROI and analysis, so it may lead to a decrease in the accuracy of analysis because of fatigue Analysis of HPs highly depends on many factors such as fixation, lab protocols, sample orientations, human expertise in tissue preparation, color variation The hardest imaging modality for applying a DL approach for the classification of cancers, and it needs high computational resources for analysis
DBT	Increases cancer detection rate Can find cancers that were entirely missed on MMs Presents a unique opportunity for AI systems to help develop DBT-based practices from the ground up. Captures a more detailed view of tissues by rotating the X-ray emitter to receive multiple images Has great capability to distinguish small lesions which may obscure the projections obtained using MMs	Time consuming and expensive because of making 3D images Lack of proper data curation and labeling Decreasing accuracy of analysis when using 2D slices instead of 3D images Looking only at 2D slices, it is still unclear whether AIModels operate better using abnormalities labeled Using bounding boxes or tightly-drawn margins of lesions DBT studies easily require more storage than MMs by order of magnitude or more.
PET	An efficient method in the analysis of small lesions Great capability to detect metastasis at different sites and organs. Checks up the entire patient for local recurrence, lymph node metastases, and distant metastases using a single injection of activity Highly recommended for patients with dense breasts or implants	Poor detection rates for small or non-invasive breast cancers Missed osteoblastic metastases showed lower metabolic activity

**Table 2:** Public datasets for different imaging modalities for breast cancer analysis.

Imaging Modality	Public Dataset	Link of Dataset	Information about Dataset
MM	BCDR	https://www.medicmind.tech/cancer-imaging-data accessed date: 25 September 2022	426 benign and 310 malignant
	IRMA	https://www.medicmind.tech/cancer-imaging-data accessed date: 25 September 2022	1865 typical cases and 932 abnormal
	MIAS	https://www.medicmind.tech/cancer-imaging-data accessed date: 25 September 2022	133 abnormal and 189 of normal class
	DDSM	https://www.medicmind.tech/cancer-imaging-data accessed date: 25 September 2022	912 benign and 784 malignant
	INBreast	http://marathon.csee.usf.edu/Mammography/Database.html accessed date: 25 September 2022	410 malignant
US	MBUD	https://www.kaggle.com/datasets/aryashah2k/breast-ultrasound-images-dataset accessed date: 25 September 2022	472 normal 278 abnormal
	OASBUD	http://bluebox.ippt.gov.pl/~hpiotrzk/ accessed date: 25 September 2022	48 benign 52 malignant
	BUSI	https://scholar.cu.edu.eg/?q=afahmy/pages/dataset accessed date: 25 September 2022	620 benign 210 malignant
	MT-small	https://www.kaggle.com/datasets/mohammedtgadallah/mt-small-dataset accessed date: 25 September 2022	200 benign 200 malignant
	UDIAT	https://datasets.bifrost.ai/info/1320 accessed date: 25 September 2022	110 benign 53 malignant
	STUHospital	https://github.com/xbhlk/STU-Hospital accessed date: 25 September 2022	42 malignant
MRI	DCE-MRI	https://mridiscover.com/dce-mri/ accessed date: 25 September 2022	559 malignant
	DWI	https://radiopaedia.org/articles/diffusion-weighted-imaging-2?lang=us accessed date: 25 September 2022	328 malignant
	RIDER	https://wiki.cancerimagingarchive.net/display/Public/RIDER+Collections accessed date: 25 September 2022	500 malignant
	DMR-IR	http://visual.ic.uff.br/dmi/ accessed date: 25 September 2022	267 normal 44 abnormal
	TCIA	https://www.cancerimagingarchive.net/ accessed date: 25 September 2022	91 malignant
HP	BreakHis	https://www.kaggle.com/datasets/ambarish/breakhis accessed date: 25 September 2022	2480 benign and 5429 malignant
	Camelyon	https://camelyon16.grand-challenge.org/Data/ accessed date: 25 September 2022	240 benign 160 malignant
	TUPAC	https://github.com/DeepPathology/TUPAC16_AlternativeLabels accessed date: 25 September 2022	50 benign 23 malignant
	BACH	https://zenodo.org/record/3632035#.Yxl8gnbMK3A accessed date: 25 September 2022	37 benign 38 malignant
	ICPR 2012	http://icpr2012.org/ accessed date: 25 September 2022	50 malignant
	IDC	https://imaging.datacommons.cancer.gov/ accessed date: 25 September 2022	162 malignant
	Wisconsin	https://archive.ics.uci.edu/ml/datasets/Breast+Cancer+Wisconsin+%28Diagnostic%29 accessed date: 25 September 2022	357 benign and 212 malignant
	DRYAD	https://datadryad.org/stash/dataset/doi:10.5061/dryad.05qfttf4t accessed date: 25 September 2022	173 malignant
	CRC	https://paperswithcode.com/dataset/crc accessed date: 25 September 2022	2031 normal 1974 malignant
	AMIDA	https://www.amida.com/index.html accessed date: 25 September 2022	23 malignant
	TCGA	https://portal.gdc.cancer.gov/ accessed date: 25 September 2022	1097 malignant
DBT	BCS-DBT	https://sites.duke.edu/mazurowski/resources/digital-breast-tomosynthesis-database/ accessed date: 25 September 2022	22,032 DBT volume from 5610 subjects (89 malignant, 112 benign, 5129 normal)

**Table 3:** The summary of the studies that used MM and DBT datasets.

Paper	Year	Task	Model	Type	Dataset	Evaluation
Agnes et al. [146]	2020	Classification	Multiscale All CNN	MM	MIAS	Acc = 96.47%
Shu et al. [156]	2020	Classification	CNN	MM	INbreast CBIS-DDSM	INbreast: Acc = 92.2% CBIS: Acc = 76.7%
Singh et al. [150]	2020	Classification	CNN	FFDM and DBT	Private	FFDM: AUC = 0.9 DBT: AUC = 0.85
Boumaraf et al. [157]	2020	Classification	DBN (Deep Belief Network)	MM	DDSM	Acc = 84.5%
Matthews et al. [158]	2021	Classification	Transfer learning based on ResNet	DBT	Private	AUC = 0.9
Zhang et al. [159]	2021	Classification	GNN (Graph Neural Network) + CNN	MM	MIAS	Acc = 96.1%
Li et al. [160]	2021	Classification	SVM (Support Vector Machine)	MM	INbreast	Acc = 84.6%
Saber et al. [161]	2021	Classification	CNN/Transfer learning	MM	MIAS	Acc = 98.87% F-score = 99.3%
Malebary et al. [162]	2021	Classification	CNN	MM	DDSM MIAS	DDSM: Acc = 97% MIAS: Acc = 97%
Li et al. [163]	2021	Classification	CNN-RNN (Recurrent Neural Network)	MM	DDSM	ACC = 94.7%, Recall = 94.1% AUC = 0.968
Ueda et al. [164]	2022	Classification	CNN	MM	Private DDSM	AUC = 0.93
Mota et al. [165]	2022	Classification	CNN	DBT	VICTRE	AUC = 0.941
Bai et al. [166]	2022	Classification	GCN (Graph Convolutional Network)	DBT	BCS-DBT Private	Acc = 84% AUC = 0.87
Zhu et al. [167]	2018	Mass Segmentation	FCN (Fully Convolutional Network) + CRF (Conditional Random Field)	MM	INbreast DDSM-BCRP	INbreast: Dice = 90.97% DDSM-BCRP: Dice = 91.3%
Wang et al. [168]	2019	Mass Segmentation	MNPNet (Multi-Level Nested Pyramid Network)	MM	INbreast DDSM-BCRP	INbreast: Dice = 91.1% DDSM-BCRP: Dice = 91.69%
Saffari et al. [169]	2020	Dense tissue Segmentation/Classification	cGAN and CNN	MM	INbreast	S: Acc = 98% C: Acc = 97.85%
Ahmed et al. [170]	2020	Tumor Segmentation/Classification	DeepLab/mask RCNN	MM	MIAS CBIS-DDSM	DeepLab: C: Acc = 95% S: MAP = 72% Mask RCNN: C: Acc = 98% S: MAP = 80%
Buda et al. [171]	2020	Lesion detection	CNN	DBT	Private	Sensitivity = 65%
Cheng et al. [172]	2020	Mass Segmentation	Spatial Enhanced Rotation Aware Net	MM	DDSM	Dice = 84.3% IOU = 73.95%
Chen et al. [173]	2020	Mass Segmentation	Modified U-Net	MM	INbreast CBIS-DDSM	INbreast: Dice = 81.64% CBIS: Dice = 82.16%
Soleimani et al. [174]	2020	Breast-Pectoral Segmentation	CNN	MM	MIAS CBIS-DDSM INbreast	MIAS: Dice = 97.59% CBIS: Dice = 97.69% INbreast: Dice = 96.39%
Al-antari et al. [175]	2020	Breast lesions Segmentation/Classification	YOLO	MM	DDSM INbreast	S: DDSM: F1-score = 99.28% INbreast: F1-score = 98.02% C: DDSM: Acc = 97.5% INbreast: Acc = 95.32%
Li et al. [176]	2020	Mass Segmentation	Siamese-Faster-RCNN	MM	INbreast BCPKUPH(private) TXMD(private)	INbreast: TP = 0.88, BCPKUPH: TP = 0.85 TXMD: TP = 0.85
Peng et al. [177]	2020	Mass Segmentation	Faster RCNN	MM	CBIS-DDSM INbreast	CBIS: TP = 0.93 INbreast: TP = 0.95
Kavitha et al. [178]	2021	Mass Segmentation/Classification	CapsNet	MM	MIAS DDSM	MIAS: Acc = 98.5% DDSM: Acc = 97.55%
Shoshan et al. [179]	2021	Lesion detection	CNN	DBT	DBTex challenge	Avg. sensitivity = 0.91
Hossain et al. [180]	2022	Lesion detection	CNN	DBT	DBTex challenge	Avg. sensitivity = 0.815
Hossain et al. [181]	2022	Lesion detection	CNN	DBT	DBTex challenge	Avg. sensitivity = 0.84
Atrey et al. [182]	2022	Breast lesion Segmentation	CNN	MM	DDSM	Dice = 65%

**Table 4:** The summary of the studies that used ultrasound dataset.

Paper	Year	Task	Model	Dataset	Evaluation
Byra et al. [204]	2019	Classification	Transfer learning based on VGG-19 and InceptionV3	OASBUD	VGG19: AUC = 0.822 InceptionV3: AUC = 0.857
Byra et al. [186]	2019	Classification	Transfer learning based on VGG 19	Private	AUC = 0.936
Hijab et al. [205]	2019	Classification	Transfer learning based on VGG16	Private	Acc = 97.4% AUC = 0.98
Zhang et al. [206]	2019	Classification	Deep Polynomial Network (DPN)	Private	Acc = 95.6% AUC = 0.961
Fujioka et al. [207]	2020	Classification	CNN	Private	AUC = 0.87
Wu et al. [208]	2020	Classification	Random Forest (RF)	Private	Acc = 86.97%
Wu et al. [209]	2020	Classification	Generalized Regression Neural Network (GRNN)	Private	Acc = 87.78% F1 score = 86.15%
Gong et al. [210]	2020	Classification	Multi-view Deep Neural Network Support Vector Machine (MDNNSVM)	Private	Acc = 86.36% AUC = 0.908
Moon et al. [195]	2020	Classification	VGGNet + ResNet + DenseNet (Ensemble loss)	SNUH BUSI	SNUH: Acc = 91.1% AUC = 0.9697 BUSI: Acc = 94.62% AUC = 0.9711
Zhang et al. [211]	2020	Classification	CNN	Private	AUC = 1
Yousef Kalaf et al. [212]	2021	Classification	Modified VGG16	Private	Acc = 93% F1 score = 94%
Misra et al. [213]	2022	Classification	Transfer learning based on AlexNet and ResNet	Private	Acc = 90%
Vakanski et al. [214]	2020	Tumor Segmentation	CNN	BUSI	Acc = 98% Dice score = 90.5%
Byra et al. [215]	2020	Mass Segmentation	CNN	Private	Acc = 97% Dice score = 82.6%
Singh et al. [216]	2020	Tumor Segmentation	CNN	Mendeley UDIAT	Mendeley: Dice = 0.9376 UDIAT: Dice = 86.82%
Han et al. [217]	2020	Lesion Segmentation	GAN	Private	Dice = 87.12%
Wang et al. [218]	2021	Lesion Segmentation	Residual Feedback Network	1-Ultrasoundcases.info and BUSI 2- UDIAT 3- Radiopaedia	1-Dice = 86.91% 2-Dice = 81.79% 3-Dice = 87%
Wang et al. [219]	2021	Segmentation	CNN	Ultrasoundcases.info BUSI STUHospital	Ultrasoundcases: Dice = 84.71% BUSI: Dice = 83.76% STUHospital: Dice = 86.52%
Li et al. [220]	2022	Tumor Segmentation + Classification	DeepLab3	Private	S: Dice = 77.3% C: Acc = 94.8%
Byra et al. [221]	2022	Mass Segmentation + Classification	Y-Net	Private	S: Dice = 64.0% C: AUC = 0.87

**Table 5:** Summary of the studies that used MRI datasets.

Paper	Year	Task	Model	Dataset	Evaluation
Ha et al. [238]	2019	Classification	CNN	Private	Acc = 70%
Ha et al. [239]	2019	Classification	CNN	Private	Acc = 88%
Fang et al. [240]	2019	Classification	CNN	Private	Acc = 70.5%
Zheng et al. [241]	2020	Classification	CNN	TCIA	Acc = 97.2%
Holste et al. [242]	2021	Classification	Fusion Deep learning	Private	AUC = 0.9
Winkler et al. [243]	2021	Classification	CNN	Private	ACC = 92.8%
Fujioka et al. [244]	2021	Classification	CNN	Private	AUC = 0.89
Liu et al. [245]	2022	Classification	Weakly ResNet-101	Private	AUC = 0.92 ACC = 94%
Bie et al. [246]	2022	Classification	CNN	Private	ACC = 92% Specificity = 94%
Jing et al. [247]	2022	Classification	U-NET and ResNet 34	Private	AUC = 0.81
Wu et al. [248]	2022	Classification	CNN	Private	Acc = 87.7% AUC = 91.2%
Verburg et al. [249]	2022	Classification	CNN	Private	AUC = 0.83
Dutta et al. [250]	2021	Tumor Segmentation	Multi-contrast D-R2UNet	Private	F1 score = 95%
Carvalho et al. [251]	2021	Tumor Segmentation	SegNet and UNet	QIN Breast DCE-MRI	Dice = 97.6% IOU = 95.3%
Wang et al. [252]	2021	Lesion Segmentation	CNN	Private	Dice = 76.4%
Nowakowska et al. [253]	2022	Segmentation of BPE area and non-enhancing tissue	CNN	Private	Dice = 76%
Khaled et al. [254]	2022	Lesion segmentation	3D U-Net	TCGA-BRCA	Dice = 68%
Yue et al. [255]	2022	Segmentation	Res_U-Net	Private	Dice = 89%
Rahimpour et al. [256]	2022	Tumor Segmentation	3D U-Net	Private	Dice = 78%
Zhu et al. [257]	2022	Lesion Segmentation/Classification	V-Net	Private	S: Dice = 86% C: Avg. AUC = 0.84
